# Attitudes Toward Entrepreneurship Education, Post-pandemic Entrepreneurial Environment, and Entrepreneurial Self-Efficacy Among University Students

**DOI:** 10.3389/fpsyg.2022.758511

**Published:** 2022-04-29

**Authors:** Jiping Zhang, Jianhao Huang, Yao Hong

**Affiliations:** ^1^Ningbo University, Ningbo, China; ^2^Dhurakij Pundit University, Lak Si District, Thailand; ^3^Shaoguan University, Shaoguan, China

**Keywords:** attitudes toward entrepreneurship education, entrepreneurial self-efficacy, university students, post-pandemic entrepreneurial environment, COVID-19

## Abstract

Currently, little is known about the mechanism of how university students’ attitudes toward entrepreneurship education (ATEE) affect entrepreneurial self-efficacy (ESE) in the post-pandemic entrepreneurial environment. Based on the existing research, this study explores the relationship between ATEE, the post-pandemic entrepreneurship environment, and ESE through a questionnaire survey. A total of 910 university students from three universities in Zhejiang Province, China participated, with an effective rate of 92.9%. The data collection focused on the period from August to December 2020. In this study, Stepwise Multiple regression analysis was used to analyze university students' ATEE and its impact on ESE, as well as the moderating effect of the post-pandemic entrepreneurial environment. The results show that the gender of university students and the entrepreneurial experience of their family members exist significant differences in their ATEE and also on their ESE. Furthermore, ATEE exert a significant and positive impact on their entrepreneurial self-efficacy, while the post-pandemic entrepreneurial environment plays a positively moderating role in this influential process.

## Introduction

Entrepreneurship education has always been an important program of colleges and universities to cultivate innovative and entrepreneurial talents. A variety of entrepreneurship courses, projects, and competitions have been emerging (Yang et al., 2021). Administrators and researchers place a larger emphasis on entrepreneurship education in colleges and universities and look forward to achieving impressive results. Since 2019, the world had experienced the COVID-19 pandemic. Due to this major crisis of public health, countries and the global economy had been hit hard (Jawad et al., 2021), resulting in rising unemployment (Rigby, 2021). Entrepreneurship has been an important means of stimulating economic recovery (Crecente-Romero et al., 2019) and also one of the effective ways how the state promotes the employment of university students. Therefore, university students' entrepreneurship plays a very crucial role in reducing unemployment and improving economic recovery in the post-epidemic period. Entrepreneurial self-efficacy (ESE) referred to an individual's confidence in fulfilling the entrepreneurial role and completing entrepreneurial tasks (Chen et al., 1998). Past studies had found that university students' ESE was a predictor of their probability of becoming entrepreneurs in the future (Chen et al., 1998) and a significant criterion for testing the achievements of entrepreneurship education (Najib et al., 2020).

Among the various influencing factors associated with university students' ESE, entrepreneurship education had been proven to have a considerable impact (Chen et al., 1998; Zhao et al., 2005; Jiang et al., 2017). Planning and Behavior Theory was referenced to argue that individual attitudes toward behavior would influence behavioral intention (Ajzen, 1991). Attitude was a way in which a person reacts to things, which can be evaluated as positive or negative reactions, and it exerted an important influence on the decision-making of human behavior (Freedman, 1984; Eagly and Chaiken, 1993). Combining the two concepts of entrepreneurship education and attitude, university students' attitudes toward entrepreneurship education (ATEE) refers to their psychological response after receiving a series of entrepreneurial education. University students' ATEEs have a significant impact on their entrepreneurial intentions (Jena, 2020). However, the correlation between ATEE and ESE needs to be further explored.

At present, most countries in the world remain in the stage of containing the novel coronavirus outbreak. While people are working hard to control the spreading of the virus, epidemic prevention and control had also brought profound changes to styles of people's learning, work, and life (Tang and Liang, 2021). These unique circumstances had created a post-pandemic entrepreneurial environment. Past studies had found that the entrepreneurial environment played an important role in entrepreneurial research. It emphasized that people's perception of the entrepreneurial environment would influence their entrepreneurial psychology and intentions (Fini et al., 2011; (Méndez-Picazo et al., 2021). The social cognitive theory suggested that there were interactions between the individual, the environment, and their behaviors. Therefore, there may be significant differences in the reactions of university students toward different degrees of perception of the post-epidemic entrepreneurial environment, resulting in the interactive relationship between the ATEE and the ESE. In other words, university students who perceive the post-epidemic entrepreneurial environment as better will show a positive ATEE and will form a higher sense of ESE. Conversely, university students who perceive the post-epidemic entrepreneurial environment as poorer will show a negative ATEE, and thus form a lower sense of ESE. To better interpret the impact of ATEE on ESE in the post-epidemic entrepreneurial environment, this study intends to carry out an empirical test.

In summary, this study not only examines the influence of university students' ATEE on ESE, but also further explores the regulatory role of the post-epidemic entrepreneurial environment on this influence. There are two major questions in this study: First, whether university students' ATEE positively affect ESE? Second, whether the post-epidemic entrepreneurial environment can effectively adjust the impact of university students' ATEE on ESE? This will enable to further understand the critical factors affecting the university students' ESE, thus providing a new direction for the effectiveness of implementing entrepreneurship education in universities, and also offering guidance significance for improving ESE of university students.

### University Students’ Attitudes Toward Entrepreneurship Education and Entrepreneurial Self-Efficacy

Lee and Wong, (2003) argued that the ATEE is the attitudes of accepting entrepreneurship, including both positive and negative ones. This study defined university students' ATEE as a psychological response after receiving a series of entrepreneurial education (Jena, 2020). Ellis (2015) believed that human attitudes were composed of three major components: cognition, emotion, and behavioral tendencies. Jena (2020) also divided university students' ATEE into cognitive components, emotional components, and behavioral tendencies. Specifically, the cognitive components included the cognition, evaluation, thought and knowledge of entrepreneurship education; the emotional component was the emotional response and feeling of students to entrepreneurship education; the behavioral tendency referred to the student's reaction or behavioral intention to entrepreneurship education. This study refers to past literature for dividing university students' ATEE into three parts: the cognitive one, the emotional one, and the behavioral tendency one (Lee and Wong, 2003; Jena, 2020). Self-efficacy referred to a person's assessment of his or her abilities to cope with situations and overcome obstacles, as well as his/her prediction about the success of future actions (Bandura, 1982). Applying self-efficacy to the field of entrepreneurship is defined as ESE. The ESE in this study referred to the strength of university students' belief in their ability to achieve entrepreneurial success by performing multiple roles and tasks and was also an important criterion for distinguishing the entrepreneurial and non-entrepreneurial (Boyd and Vozikis, 1994; Krueger and Brazeal, 1994).

Empirical studies had shown that university students' ATEE significantly positively affected entrepreneurial intentions (Jena, 2020), and so did ESE (Wu et al., 2022), hence this study inferred that university students' ATEE might significantly positively affect ESE. Past studies had also found that university students' ATEE had a positive impact on their entrepreneurial intentions (Lee and Wong, 2003). Through the research of Liñán et al. (2011), it was found that active participation in entrepreneurship education could enhance university students' entrepreneurial confidence by improving their skills, broadening their horizons, and deepening their beliefs, eventually stimulating their ESE. Entrepreneurship education can also help university students gain entrepreneurial knowledge (Dohse and Walter, 2012). Practical courses in entrepreneurship education for university students can teach them entrepreneurial knowledge applicable to master new technologies and markets information (Shane, 2004). Similarly, through developing cognitive skills at the individual level, university students can distinguish between valuable and available information, identify entrepreneurial opportunities, and improve their entrepreneurial skills, thereby enhancing their confidence in entrepreneurial success and gaining ESE (Miralles et al., 2016). Based on these, this study proposes the hypothesis that:

*Hypothesis* 1: University students’ ATEEs can significantly affect their ESEs.

### Moderating Role of the Post-pandemic Entrepreneurial Environment

The entrepreneurial environment has been playing an important role in the development of entrepreneurship. Shane (2004) argued that the entrepreneurial environment for university students involves three factors: economy, politics, and society. As a consequence of the global pandemic, the economic, political, and social environments have substantially changed and people have become more inclined to using the Internet for learning, shopping, communicating, and living. It has been revealed that the entrepreneurial environment is a key variable to ESE, which is therefore often applied in other areas of entrepreneurial research. For example, Kim and Kim (2016) found that the entrepreneurial environment moderated the relationship between entrepreneurial orientation and firm performance. Wang et al. (2021) pointed out that the entrepreneurial environment regulated the relationship between individuals' entrepreneurial orientation and value congruence. In an empirical study of freelancers, Huđek et al. (2021) found that the entrepreneurial environment was a key factor to success in their career, and indeed, freelancing could also be deemed as a form of self-employment. Therefore, the better the entrepreneurial environment, the more likely freelancers will succeed.

Bandura proposed a social cognitive theory, which emphasized the “individual–environment–behavior” interaction (Bandura, 1986). The concept of this theory is based on the studies of human information processing and human cognitive activities. The social cognitive theory suggests that individuals collect information from their surroundings before interpreting and processing it by their own cognitive and emotional or other attributes to make new social decisions and create new mental states (Fiske and Taylor, 1991; Robinson and Marino, 2015). The social environment where one lives normally exerts an important effect on applying his or her resources to enhance self-prediction, operation, or volitional control (De Carolis and Saparito, 2006). The social environment also affects one’s self-regulatory system, that is, self-efficacy (Hmieleskik and Baron, 2009). Gedajlovic et al. (2013) also found that individual ESE is not only directly affected by individual factors and environmental factors, but also influenced by the interaction between these two types of factors. Packham et al. (2010) compared three different countries, Germany, Poland, and France, and found that under different entrepreneurial environments, university students had different attitudes toward entrepreneurship. Kim and Kim (2016) argued that the entrepreneurial environment usually played a moderating role in the entrepreneurial field. According to the theory of social cognition (Bandura, 1986), it was considered that the attitudes of university students toward entrepreneurship education could be regarded as an individual factor, and the post-epidemic entrepreneurial environment could be regarded as an environmental factor. ESE may not only be directly affected by university students' ATEE and the post-epidemic entrepreneurial environment, but also by the interaction between the two of them. Therefore, this study proposes:

*Hypothesis* 2: The post-pandemic entrepreneurial environment performs a moderating role in the relationship between university students’ ATEE and ESE.

## Methodology

### Research Framework

**Figure 1:**
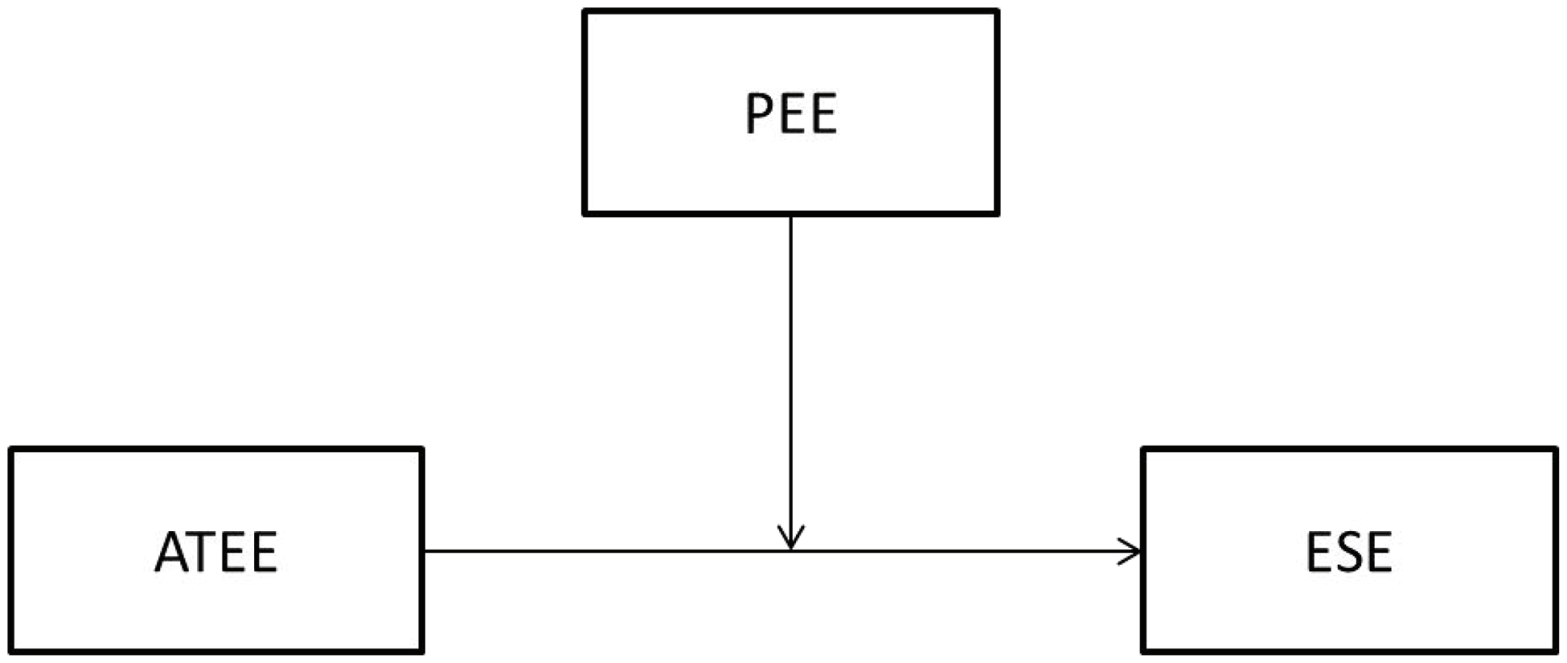
Research framework. ATEE means attitudes toward entrepreneurship education; PEE means post-pandemic entrepreneurial environment; ESE means entrepreneurial self-efficacy.

In this study, the relationship between ATEE and entrepreneurial self-efficacy (ESE) and the moderating effect of the Post-pandemic Entrepreneurial Environment (PEE) in this relationship were investigated using ATEE as the independent variable, ESE is the dependent variable, and PEE as the moderator (Figure 1).

### Data Collection and Procedures

Each questionnaire involved in this study consists of four sections, namely, basic information about the participants, a scale of university students’ ATEE, a scale of their ESE, and a scale of post-pandemic entrepreneurial environment. Two English majors were asked to conduct translation and back translation for each of the three scales from the original questionnaire to narrow down cross-cultural differences.

Before the formal test, this study applied the convenience sampling method to select 119 volunteers from a comprehensive university in Zhejiang Province to conduct the pilot test. According to their feedback and the reliability and validity analysis of the pilot test data, some items that did not meet the standard were deleted, and a formal questionnaire was finally formed. This study and these questions do not pose any potential risks to participants. The study applied a 5-point Likert scale to measure each item, ranging from 1 to 5, with 1 meaning totally disagree and 5 meaning totally agree.

This study conducted anonymous surveys on ATEEs, PEEs, and ESEs of three universities respectively. Three universities in Zhejiang Province were selected as the target, which are provincial model ones for entrepreneurship education with a relatively integral major setup. The major considerations for this choice are outlined below. First, the three universities offer a comprehensive popular course of entrepreneurship “Career Planning and Entrepreneurship” for most of their junior students, while some of their senior students receive a hierarchical and categorical entrepreneurship education with elective courses, such as “Fundamentals of Entrepreneurship for University Students,” in addition to entrepreneurship projects, competitions, and practices. Second, all three universities are located in Zhejiang Province, a relatively economically developed area in China with a solid humanistic foundation and strong policy support for entrepreneurship. Finally, Zhejiang Province implemented a good control program against the COVID-19 pandemic so that people returned to work and school quite early. These are favorable factors for the creation of an entrepreneurial environment. Therefore, these three universities have been selected for this study, and their samples are both typical and pioneering in the sense of entrepreneurship education.

In the formal test, convenience sampling method was adopted to sample students from different majors of the above three universities in Zhejiang Province of China. First, we contacted the teachers responsible for entrepreneurship in universities and selected 1,050 students as the subjects of the questionnaire. Second, we conducted data collection. Data collection was mainly concentrated between August and December 2020. Participants were informed by trained researchers that participation was voluntary and anonymous and that data collected were protected by the Declaration of Helsinki (Goodyear et al., 2007). The researchers distributed and collected the online questionnaires during the students' class time. It took 10 minutes to complete the questionnaires to ensure the quality of the questionnaire answers. A total of 980 questionnaires were recovered, and the participation rate was 93.3%. Finally, we performed preliminary screening and sorting of the data. Since the online questionnaire used in this study has a missing answer prompt function, it can only be submitted after filling in all the items, so there will be no missing answers. The distribution of the average answering time of the questionnaire is 3 minutes and 5 seconds. The samples with too short answering time are regarded as invalid samples. In this study, 1 minute and 3 seconds are regarded as invalid samples. Finally, 910 valid samples were obtained, and the effective rate was 92.9%.

### Participants

Among the respondents, 220 were male and 690 were female students; The subjects of this study were university students ranging in ages from 18 to 24 years old. 560 people were juniors, and 350 people were at the undergraduate level and above. 240 had family members with experiences in entrepreneurship and 670 did not. In terms of receiving entrepreneurship education, 67.3% of the students had attended entrepreneurship courses, 62% had attended entrepreneurship lectures, 16% had participated in entrepreneurship competitions or projects, 16.8% had participated in entrepreneurship practices, and 12.1% had participated in other forms of entrepreneurship education.

### Ethics Statement

This study was approved by the Ethics Committee of Ningbo University and the test was conducted with the informed consent of the participants.

### Measures

#### Background Variables for University Students

This part of the questionnaire focuses on participants’ gender, major, grade, educational background, family members with or without entrepreneurship experience, and the form of entrepreneurship education they have participated in.

#### University Students’ Attitudes Toward Entrepreneurship Education

This study adopts a revised version of the ATEE scale created by Jena, Cronbach’s α for the cognitive component, affective component, behavioral component, were 0.76, 0.79, 0.81 respectively, AVE values for the three components were 0.69,0.72,0.67 separately. CR values for CA, AA, BA, were 0.89,0.89,0.83 individually, which all showed good validity and reliability (Jena, 2020). The research questions were integrated into a 5-point Likert scale. The scale was divided into three components, namely, the cognitive one, the affective one, and the behavioral one respectively. Items that measured the cognitive component included "The entrepreneurship education courses I took in university taught me sufficient knowledge," "The entrepreneurship courses in university taught me the ability to identify business opportunities" and so on. Items that assessed the affective component consisted of "I'm glad I had an entrepreneurship education at my university," "My university entrepreneurship education encouraged me to start a business after graduation," etc. Items that addressed the behavioral component were composed of "I want to start a business after finishing my studies," "I would love to start my own business and work for myself" and else. Exploratory Factor Analysis was carried out on the pilot test data. When the factor loading was less than 0.5 or assigned to two dimensions simultaneously, these items were deleted. And there were 13 items left on the scale after deletion. The results showed that KMO=0.883, Bartlett's Test of Sphericity value was significant (p < 0.001). Kaiser and Rice (1974) proposed that data was suitable for Exploratory Factor Analysis when KMO>0.8 and Bartlett's Test of Sphericity value was significant (p < 0.05). Therefore, this study adopted the varimax rotation to conduct an Exploratory Factor Analysis on the scale of university students’ ATEE. The rotated component Matrix displayed that 13 factors with eigenvalues greater than 1 were generated eventually. The factor loadings of the 13 factors ranged from 0.578 to 0.892, which met the criterion that they should be greater than 0.3 (Zaltman and Burger, 1975). In addition, the cumulative explained variance was 77.333%. This indicated that the scale presented good validity. Cronbach's α of each dimension was as follows: 0.95 for the cognitive component, 0.793 for the emotional component, =0.864 for the behavioral tendency component, and 0.932 for the total scale of university students’ ATEE, respectively, indicating good reliability as well (Nunally, 1978).

#### Entrepreneurial Self-Efficacy

This scale, developed by Wilson et al. (2007), exhibited good reliability and validity (Cronbach's α=0.91; AVE=0.59; CR=0.87)in the study by Rajib and Niladri (2020). It consists of 5 items, such as "I am good at problem-solving," "I am good at money management" and so on. Item analysis focuses on the pilot test data, and further screens the topics afterward, with no need to delete questions. Because this scale is one-dimensional, the results of Exploratory Factor Analysis can only generate one factor with an eigenvalue greater than 1. The factor loading was between 0.739 and 0.864, and the cumulative explained variance ratio was 64.76%. The validity of the scale was also good. Cronbach's α of the ESE Scale was 0.861, hence the reliability of the scale was good too (Cronbach, 1951).

#### Post-pandemic Entrepreneurial Environment

This scale was developed by Jena (2020) and showed good reliability and validity (Cronbach's α= 0.72; AVE=0.69; CR=0.84). It is composed of four items using a 5-point scale score. According to the characteristics of the post-epidemic environment, 7 items were added to the scale by Jena, and 2 experts were invited to evaluate the scale, to conduct item analysis on pilot test data, to further screen the items, and to delete certain items independently. The final scale includes 7 items, such as "China is a very suitable country for entrepreneurship," "The epidemic has been effectively controlled in China" and so on. Because this scale was one-dimensional, the results of Exploratory Factor Analysis could only generate one factor with an eigenvalue greater than 1, the factor loading was between 0.685 and 0.867, and the cumulative explained variance ratio was 60.2%. The validity of the scale was proven good. Cronbach's α of the Post-pandemic Entrepreneurial Environment Scale was 0.889, which indicated that the scale owned good reliability.

### Reliability and Validity of Formal Test Data

#### University Students’ Attitudes Toward Entrepreneurship Education

Confirmatory Factor Analysis (see Table 1) was performed on the formal test data of ATEE, and the results showed: RMR=0.031 (less than 0.08), GFI=0.849 (close to 0.9), NFI=0.923 (more than 0.9), IFI=0.927 (more than 0.9), TLI=0.908 (greater than 0.9), CFI=0.927 (greater than 0.9), SRMR=0.036 (less than 0.08), indicating that the measurement model fitted the observed data well (McDonald and Ho, 2002). The Average Variance Extracted and Composite Reliability of each variable of the scale are as follows: CR=0.89 for cognitive component, CR=0.756 for affective component, CR=0.854 for behavioral tendency component, which are all greater than the reference value of 0.6 (Anderson and Gerbing, 1988), thus the standards are compliant. There are three dimensions of the scale on university students' entrepreneurship education, among which the AVE=0.537 for the cognitive component, the AVE=0.511 for the emotional component, and the AVE=0.662 for the behavioral disposition component, indicating good convergence validity. Cronbach's α values of each dimension are as follows: 0.962 for the cognitive component; 0.846 for the emotional component, and 0.91 for the behavioral tendency component. Cronbach's α=0.965 of the total scale of university students' ATEE indicates good reliability (Nunally, 1978).

**Table 1:** Confirmatory factor analysis and reliability analysis.

Variable	Item	Factor Loading	AVE	CR	Cronbach's α
Cognitive Component	I enjoyed lectures on entrepreneurship as offered in the university	0.822	0.537	0.89	0.962
	Lectures on entrepreneurship I received in university have increased my interest to pursue an entrepreneurial career	0.825			
	Due to the university’s/college’s entrepreneurship education program, I now have skills to can create a new business	0.785			
	University/college entrepreneurship courses have enabled me to identify business related opportunities.	0.673			
	Entrepreneurship education program of the university/college has taught me to perform feasibility studies	0.651			
	The university’s/college’s entrepreneurship courses have stimulated my interest in entrepreneurship	0.694			
	Overall, I am very satisfied with how entrepreneurship education program is being taught in my university	0.652			
Affective Component	I am happy to have had entrepreneurship education in my university	0.825	0.511	0.756	0.846
	Entrepreneurship education I received in university/college has encouraged me to venture into entrepreneurship after graduation	0.67			
	My university entrepreneurship staffers help students to meet successful entrepreneurs who provide motivation to students to become entrepreneurs.	0.635			
Behavioral Component	I would want to be an entrepreneur after my study	0.834	0.662	0.854	0.91
	The idea to become an entrepreneur and work for my self is appealing to me	0.868			
	I really consider self-employment as something very important	0.733			
Post-pandemic Entrepreneurial Environment	China is an excellent country to start a business	0.758	0.648	0.928	0.959
	China's local government supports entrepreneurs	0.83			
	China's epidemic has been effectively controlled	0.76			
	China's economic environment stabilizes	0.838			
	People's way of thinking and lifestyle have changed	0.821			
	Government provides more entrepreneurial support	0.848			
	Post-pandemic environment brings opportunities for entrepreneurship	0.776			
Entrepreneurial Self-efficacy	I am good at problem-solving	0.817	0.673	0.911	0.944
	I am good at money management	0.804			
	I can often get others to agree with me	0.858			
	I like being a leader	0.796			
	I'm good at making decisions	0.824			

#### Post-epidemic Entrepreneurial Environment

Confirmatory Factor Analysis was performed on the formal test data of PEE, and the results showed: RMR=0.029 (less than 0.08), NFI=0.893 (close to 0.9), IFI=0.895 (close to 0.9), TLI=0.842 (close to 0.9), CFI=0.895 (close to 0.9), RMSEA=0.062 (less than 0.08), and SRMR=0.043 (less than 0.08), indicating that the measurement model basically fitted the observed data (McDonald and Ho, 2002). The Average Variance Extracted and Composite Reliability of the scale were as follows: CR=0.928, AVE=0.648, revealing good convergence validity. Cronbach's α=0.959 for the post-pandemic entrepreneurial environment, indicating good reliability.

#### Entrepreneurial Self-Efficacy

Confirmatory Factor Analysis was performed on the formal test data of ESE, and the results showed: RMR=0.011 (less than 0.08), GFI=0.983 (more than 0.9), NFI=0.991 (more than 0.9), IFI=0.992 (more than 0.9), TLI=0.984 (more than 0.9), CFI=0.992 (greater than 0.9), SRMR=0.013 (less than 0.08), showing that the measurement model fitted the observed data well (McDonald and Ho, 2002). The Average Variance Extracted and Composite Reliability of the scale were as follows: CR=0.911, AVE=0.673, indicating good convergence validity. Cronbach's α=0.944 of the Entrepreneurial Self-Efficacy scale, revealing good reliability.

### Data Analysis

The data analysis and statistics in this study fall into the following steps: First, SPSS and AMOS are used to test the reliability and validity of the scale of university students’ attitudes toward entrepreneurship education, the scale of post-pandemic entrepreneurial environment, and the scale of entrepreneurial self-efficacy; next, a correlation analysis is made to explore the correlations among the three main variables; finally, if the correlations among the three variables were significant, a hierarchical regression analysis would be applied to explore the mechanisms of action between the variables and the environment moderating effect of post-pandemic entrepreneurship.

### Common Method Biases Test

According to the result of the confirmatory factor analysis of the one-factor model in this study, however, the one-factor model is found not to fit the data well (*χ*^2^ = 7733.281, df = 275, GFI = 0.481, RMSEA = 0.173); the hypothetical model (*χ*^2^ = 2539.988, df = 265, GFI = 0.803, RMSEA = 0.097) significantly outperforms the one-factor model (Δ*χ*^2^ = 5193.293, Δdf = 10, *p* < 0.001). Although the above tests fail to rule out the threat of common method biases, they have provided evidence that the problem of common method biases in this study is not serious.

## Results

### Descriptive Statistics and Correlation Analysis

According to Table 2, The three variables have a minimum value of 1 and a maximum value of 5. The means, standard deviations, and correlation matrix between variables in the study are shown in Table 2. University students’ attitudes toward entrepreneurship education are moderately high (M = 3.606, SD = 0.790); university students are relatively optimistic toward the post-pandemic entrepreneurial environment (M = 3.871, SD = 0.729); and their entrepreneurial self-efficacy is slightly low (M = 3.486, SD = 0.813). All three variables in this study are continuous variables. Because a Pearson correlation is a parametric test that is appropriate when the two variables are continuous, Pearson correlation analysis is used to test the degree of correlation between variables. The correlation analysis reveals a significant positive correlation between all variables in this study (0.671-0.770).

**Table 2:** Correlation analysis of attitudes toward entrepreneurship education, post-pandemic entrepreneurial environment, and entrepreneurial self-efficacy.

Variable	Min	Max	M	SD	ATEE	PEE	ESE
ATEE	1	5	3.606	0.790	0.748	–	–
PEE	1	5	3.871	0.729	0.762^***^	0.805	–
ESE	1	5	3.486	0.813	0.770^***^	0.671^***^	0.820

*The diagonal elements in bold are square roots of AVE. ATEE means attitudes toward entrepreneurship education; PEE means post-pandemic entrepreneurial environment; ESE means entrepreneurial self-efficacy*.

*** *p* *< 0.001*.

### Analysis of the Differences in Attitudes Toward Entrepreneurship Education and Entrepreneurial Self-Efficacy by Gender and Family Members With or Without Entrepreneurship Experience

The results of t-tests on the independent samples (Tables 3 and 4) have the following implications: There is no significant gender difference in attitudes toward entrepreneurship education (*t* = 1.954, *p* = 0.051); male students perceive the post-pandemic entrepreneurial environment significantly higher than do female students (*t* = 2.165, *p* < 0.05); male students have significantly higher entrepreneurial self-efficacy than do female students (*t* = 4.328, *p* < 0.001); students whose family members have entrepreneurship experience have significantly higher attitudes toward entrepreneurship education than students whose family members have no such experience (*t* = 3.447, *p* < 0.01); entrepreneurial self-efficacy is significantly higher among students whose family members have entrepreneurship experience than among students whose family members have no such experience (*t* = 4.035, *p* < 0.001); students whose family members have entrepreneurship experience perceive the post-pandemic entrepreneurial environment significantly higher than students whose family members have no such experience (*t* = 3.124, *p* < 0.01).

**Table 3:** Descriptive information of the variables in the male and female groups.

Variables	Gender	*n*	M (SD)	*t*	*η* ^2^
ATEE	Male	220	3.697 (0.805)	1.954	0.004
	Female	690	3.577 (0.784)		
PEE	Male	220	3.69 (0.835)	2.165^*^	0.005
	Female	690	3.42 (0.795)		
ESE	Male	220	3.963 (0.761)	4.328^***^	0.02
	Female	690	3.841 (0.717)		

*η*^2^ *means partial Eta-squared. ATEE means Attitudes Toward Entrepreneurship Education; PEE means Post-pandemic Entrepreneurial Environment; ESE means Entrepreneurial Self-Efficacy*.

* *p* *<0.05;*

*** *p* *<0.001*.

**Table 4:** Descriptive information on variables of family members with or without entrepreneurial experience.

Variables	FME	*n*	M (SD)	*t*	*η* ^2^
ATEE	with	239	3.756(0.788)	3.447^*^	0.013
	without	671	3.552(0.784)		
PEE	with	239	3.996(0.741)	3.124^*^	0.011
	without	671	3.826(0.720)		
ESE	with	239	3.666(0.804)	4.035^***^	0.018
	without	671	3.421(0.806)		

*η*^2^ *means partial Eta-squared; ATEE means attitudes toward entrepreneurship education; PEE means post-pandemic entrepreneurial environment; ESE means entrepreneurial self-efficacy; and FME means family members with or without entrepreneurial experience*.

* *p* *< 0.05;*

*** *p* *< 0.001*.

### Regression Analysis and Moderating Effect Examination of University Students’ Attitudes Toward Entrepreneurship Education and Entrepreneurial Self-Efficacy

Stepwise multiple regression analysis was conducted to the study. Assumption of regression analysis was tested including Independence, Normality, and Homogeneity of Variables. First, having autocorrelation means the scores can be repeating patterns or scores of a sample are not independent. Durbin–Watson value of 1.970 indicated no autocorrelation as Durbin Watson test value between 1.5-2.5 denotes no autocorrelation (Tabachnick and Fidell, 2001). Second, The skewness absolute values for the 25 items were between.028 and 0.875, and the kurtosis absolute values for the 25 items were between 0.01 and 1.156. The results satisfied the standards of the absolute value for skewness < 2 and kurtosis < 7 (Curran et al., 1996). The research data satisfied normal distribution. Therefore, the three study variables are in line with normality. Finally, a scatter plot is used to test the problem of homogeneity. Linearity was tested using both statistic and scatterplot. The values for linearity and deviation from linearity were all significant indicating linearity. Scatterplot showed the residual means were on the same straight line; therefore, linearity and homoscedasticity assumption were met. The data satisfy the homogeneity assumption.

Therefore, this study used multiple regression analysis to examine whether university students’ attitudes toward entrepreneurship education affect entrepreneurial self-efficacy and whether the post-pandemic entrepreneurial environment has a moderating effect on this, after controlling for demographic variables (gender and family members with or without entrepreneurship experience) that can also have an effect on university students’ attitudes toward entrepreneurship education and entrepreneurial self-efficacy. Convert the control variables into dummy variables (1 = male, 0 = female; 1 = family members with entrepreneurship experience, 0 = without entrepreneurship experience), turn the control variables into independent variables (Treat entrepreneurial self-efficacy as a dependent variable.), and conduct a linear regression analysis;

Step 1: Using the forced entry method, put the attitudes toward entrepreneurship education into independent variables, and conduct regression analysis to examine the effect of attitudes toward entrepreneurship education on entrepreneurial self-efficacy, the results are as Model 1 (Table 5);

**Table 5:** Regression Analysis on the Moderating Effect of Attitudes toward Entrepreneurship Education on Entrepreneurial Self-efficacy and Post-pandemic Entrepreneurial Environment.

Variables	ESE			
	Model 1	Model 2	Model 3	VIF
Gender (Male)	0.094^***^	0.090^***^	0.088^***^	1.007
FME (with)	0.049^*^	0.045^*^	0.043^*^	1.016
ATEE	0.758^***^	0.611^***^	0.598^***^	2.441
PEE		0.194^***^	0.210^***^	2.463
ATEE*PEE			0.060^**^	1.032
F	459.094^***^	367.572^***^	298.146^***^	
R^2^	0.603	0.619	0.623	
ΔR^2^	-	0.016	0.004	

*ATEE means Attitudes Toward Entrepreneurship Education; PEE means Post-pandemic Entrepreneurial Environment; ESE means Entrepreneurial Self-Efficacy; FME means Family Members with or without Entrepreneurial experience. Gender is encoded as a dummy variable in the model: 1 = male, 0 = female. FME is encoded as a dummy variable in the model: 1 = with, 0 = without. The regression coefficients are standardized regression coefficients*.

* *p* *< 0.05;*

** *p* *< 0.01;*

*** *p* *< 0.001*.

Step 2: Use moderating variables of the post-pandemic entrepreneurial environment as third-level independent variables, and conduct the regression analysis, the results are as Model 2 (Table 5);

Step 3: By calculating the variables to obtain the results of the interaction term, standardize the interaction term as the fourth-level independent variable, and then and conduct the regression analysis including this variable to examine the impact of interaction on entrepreneurial self-efficacy. The results are as Model 3 (Table 5).

Finally, Create Table 5. As shown in the column “Model 1” of Table 4, university students’ attitudes toward entrepreneurship education positively predict entrepreneurial self-efficacy (*β* = 0.758, *p* < 0.001), indicating the higher university students’ attitudes toward entrepreneurship education, the higher their entrepreneurial self-efficacy. Therefore, Hypothesis 1 is supported.

As shown in the column “Model 3” of Table 4, the interaction between university students’ attitudes toward entrepreneurship education and the post-pandemic entrepreneurial environment significantly and positively predicts entrepreneurial self-efficacy (*β* = 0.060, *p* < 0.001). The variance inflation factor (VIF) is used as a pointer to test for multicollinearity. And the VIF values are all less than 10, which mean that there is no significant multicollinearity problem between the variables. Therefore, it can be concluded from Model 3 that the post-pandemic entrepreneurial environment performs a positive moderating role in the influence of attitudes toward entrepreneurship education on entrepreneurial self-efficacy. Therefore, Hypothesis 2 is supported.

**Figure 2:**
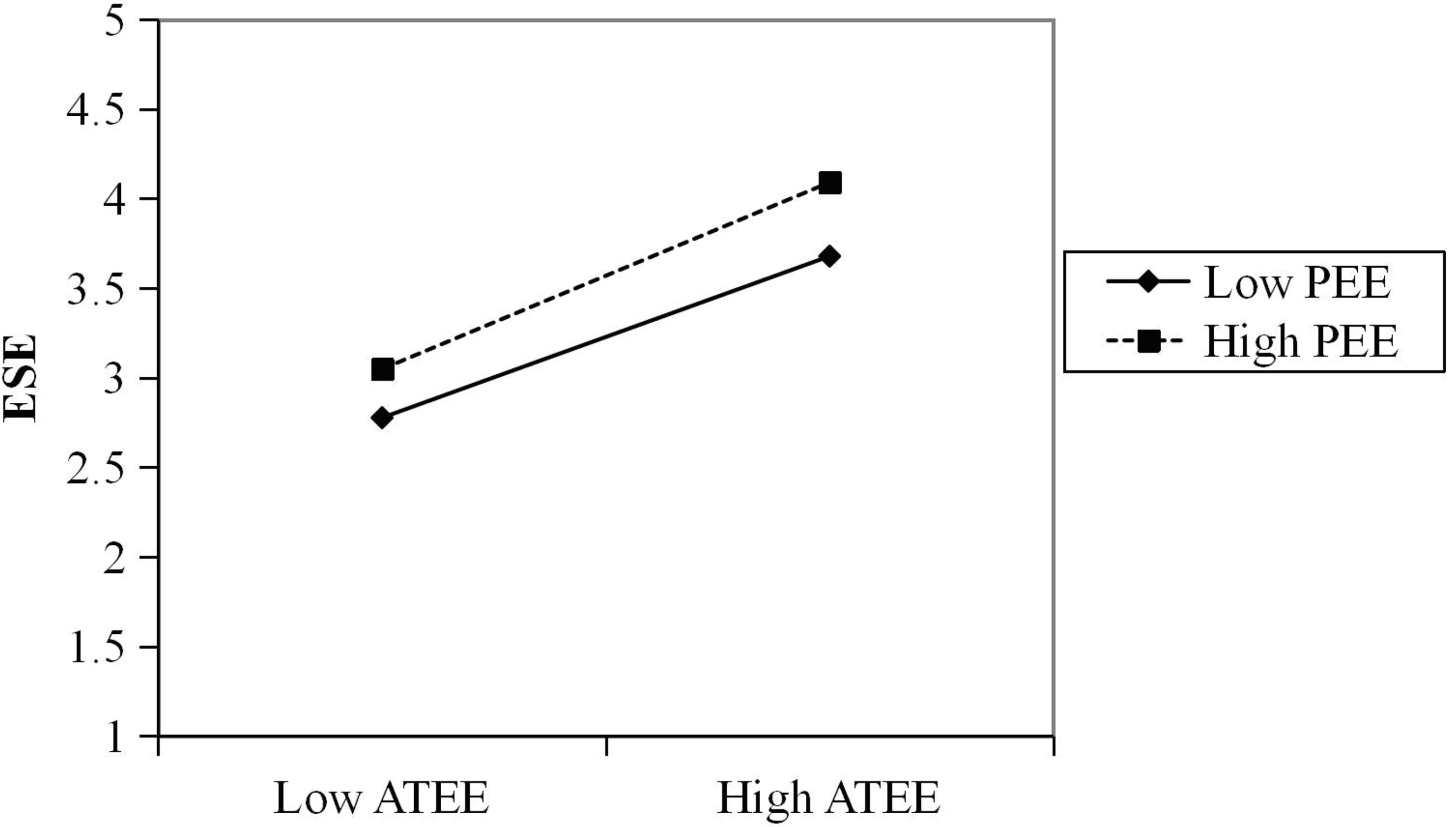
Moderating role of post-pandemic entrepreneurial environment between attitudes toward entrepreneurship education and entrepreneurial self-efficacy. ATEE means attitudes toward entrepreneurship education; PEE means post-pandemic entrepreneurial environment; ESE means entrepreneurial self-efficacy.

To further examine how the post-pandemic entrepreneurial environment moderates the impact of university students’ attitudes toward entrepreneurship education on ESE, an interaction diagram is drawn between high and low scores in the post-pandemic entrepreneurial environment, that is, one standard deviation above and one standard deviation below the mean score of the post-pandemic entrepreneurial environment, respectively. Figure 2 below visualizes how the post-pandemic entrepreneurial environment moderates the impact of university students’ attitudes toward entrepreneurship education on entrepreneurial self-efficacy. The results show that the post-pandemic entrepreneurial environment moderates the relationship between university students’ entrepreneurial attitudes and entrepreneurial self-efficacy. Specifically, the attitudes toward entrepreneurship education positively predict entrepreneurial self-efficacy for university students who score higher on the perceived post-pandemic entrepreneurial environment.

## Discussion and Conclusion

### Theoretical Contributions

First off, the findings of the study indicate that male university students have a stronger sense of entrepreneurial self-efficacy than do female university students. The findings are in line with the study by Pelegrini and Moraes (2021). Similarly, Veljkovic et al. (2019) found that female students majored in MBA have lower entrepreneurial self-efficacy and entrepreneurial intentions than do male students. This may be related to gender stereotypes acquired by individuals through their social learning: Whereas men are socially expected to dominate the source of household finances, be more capable of solving problems, and participate in competitions, women are expected to be quiet and considerate of others and take care of children. In addition, this study also finds that university students whose family members have entrepreneurship experience have a stronger sense of entrepreneurial self-efficacy than those whose family members have no such experience. This is consistent with the findings of previous studies (Krueger, 1993; Carr and Sequeira, 2007; Veljkovic et al., 2019). This difference may be due to the fact that university students are influenced by their family members who have started a company, for example, family members may provide them with financial or moral resources, a process of osmosis where the personal traits and behaviors of family members inadvertently influence these students, making them more confident in starting a business, more inclined to take risks, and more entrepreneurial. All these qualities contribute to a high level of entrepreneurial self-efficacy.

Second, the findings indicate that university students’ attitudes toward entrepreneurship education have a positive and significant effect on their entrepreneurial self-efficacy. That is, the higher the university students’ attitudes toward entrepreneurship education, the higher their entrepreneurial self-efficacy. This is in line with the previous research (Jena, 2020; Roy and Das, 2020). Entrepreneurship education activities are a specific program for students to foster higher attitudes toward entrepreneurship. The results of previous studies also prove that entrepreneurship education plays an important role in promoting entrepreneurship (Paco et al., 2015). Therefore, as university students foster higher attitudes toward entrepreneurship education, they gain more entrepreneurial cognition; as they develop stronger interest in entrepreneurship, they become more willing to engage in entrepreneurial activities. Thus, a virtuous cycle is created that promotes students’ entrepreneurial self-efficacy.

Third, the findings show that the post-pandemic entrepreneurial environment positively moderates the relationship between attitudes toward entrepreneurship education and entrepreneurial self-efficacy. This means the higher the score of perceived post-pandemic entrepreneurial environment, the more significant the positive effect of students’ attitudes toward entrepreneurship education on entrepreneurial self-efficacy. The results of this study may partially validate the social cognitive theory, which emphasizes that human behavioral intentions are influenced by the interaction between individual and environmental factors (Bandura, 1986). In this study, students’ attitudes toward entrepreneurship education can be regarded as an individual factor and the post-pandemic entrepreneurial environment as an environmental factor, and the interaction between the two effectively enhances students’ entrepreneurial self-efficacy. In order to minimize the spread of the pandemic in the post-pandemic entrepreneurial environment, people try to get adapted to the lifestyle featuring the Internet economy, such as online shopping and online learning, and their way of thinking has changed dramatically, which is both a challenge and an opportunity. As a result of the pandemic, many real economies have experienced a severe downturn, while the online economy is in a burgeoning growth. University students are more prepared with information technology and more adapted to the Internet-based lifestyle, so they are supposed to be more confident in starting their own business in the post-pandemic environment.

### Practical Implications

First, it is suggested that entrepreneurship education in universities allow for the individual differences between students. On the one hand, entrepreneurship education should allow for gender differences. On the other hand, entrepreneurship education should allow for the differences in resource base of students’ families.

Second, we suggest that entrepreneurship education in universities focus on students’ attitudes toward it. This suggestion can be implemented in the following three aspects: Firstly, in the cognitive aspect, the teaching contents covered in entrepreneurship education should be more inclusive of the instruction of basic common sense about entrepreneurship, entrepreneurial expertise training courses, and new paths for students to practice entrepreneurship, so that an all-round entrepreneurship education system can be built; secondly, in the emotional aspect, entrepreneurship education should be staffed with an outstanding teaching faculty that demonstrate good teaching styles and personal charms, keep track of students’ satisfaction with instructors and courses, and focus on students’ interest in entrepreneurship courses; finally, in the aspect of behavior tendency, students’ attitudes toward entrepreneurship education in universities can be promoted by vivifying entrepreneurship courses, so that students are more willing to attend and learn more from the classes.

Third, it is therefore recommended that the government further optimize the post-pandemic entrepreneurial environment. The government can formulate entrepreneurship incentive policies to create better platforms and favorable conditions for entrepreneurs, such as tax breaks for university students who are ready to start their own business and free business sites for entrepreneurship through university business parks.

### Limitations and Future Research Directions

Overall, this study has explored the relationship between university students’ attitudes toward entrepreneurship education and entrepreneurial self-efficacy, and the moderating effect of the post-pandemic entrepreneurial environment on their relationship. The findings have confirmed the positive predictive effect of university students’ attitudes toward entrepreneurship education and entrepreneurial self-efficacy, and revealed the positive moderating effect of the post-pandemic entrepreneurial environment on their relationship, carrying certain implications on how the government and universities can improve their entrepreneurial self-efficacy in the post-pandemic era. Nevertheless, we should also admit some limitations of this study. First, this study has adopted a cross-sectional design so that future longitudinal research can confirm the causal relationships in this study. Second, the samples in this study have been collected from exemplary universities in innovation and entrepreneurship education, and it is expected that the type of universities and sample size can be expanded in subsequent studies to obtain more evidence. Finally, this study overlooks the sociocultural factors in the entrepreneurial process (Vuong, 2016; Vuong et al., 2018, 2020), and their influences on entrepreneurial intentions will be discussed in subsequent research.

## Data Availability Statement

The raw data supporting the conclusions of this article will be made available by the authors, without undue reservation.

## Ethics Statement

The studies involving human participants were reviewed and approved by the Human Ethics Committee of the Ningbo University. The patients/participants provided their written informed consent to participate in this study. Written informed consent was obtained from the individual(s) for the publication of any potentially identifiable images or data included in this article.

## Author Contributions

JZ designed the study, analyzed the data, and drafted the manuscript. JH assisted in analyzing and interpreting the data and participated in the revision of the manuscript. YH participated in the revision of the manuscript. All authors contributed to the article and approved the submitted version.

## Conflict of Interest

The authors declare that the research was conducted in the absence of any commercial or financial relationships that could be construed as a potential conflict of interest.

## Publisher’s Note

All claims expressed in this article are solely those of the authors and do not necessarily represent those of their affiliated organizations, or those of the publisher, the editors and the reviewers. Any product that may be evaluated in this article, or claim that may be made by its manufacturer, is not guaranteed or endorsed by the publisher.
